# A G-Band Broadband Continuous Wave Traveling Wave Tube for Wireless Communications

**DOI:** 10.3390/mi13101635

**Published:** 2022-09-29

**Authors:** Yuan Feng, Xingwang Bian, Bowen Song, Ying Li, Pan Pan, Jinjun Feng

**Affiliations:** National Key Laboratory of Science and Technology on Vacuum Electronics, Beijing Vacuum Electronics Research Institute, Beijing 100015, China

**Keywords:** G-band broadband amplifiers, traveling wave tubes, folded waveguide

## Abstract

Development of a G-band broadband continuous wave (CW) traveling wave tube (TWT) for wireless communications is described in this paper. This device provides the saturation output power over 8 W and the saturation gain over 30.5 dB with a bandwidth of 27 GHz. The maximum output power is 16 W and the bandwidth of 10 W output power is 23 GHz. The 3 dB bandwidth is greater than 12.3% of f_c_ (center frequency). The gain ripple is less than 10 dB in band. A pencil beam of 50 mA and 20 kV is used and a transmission ratio over 93% is realized. The intercept power of the beam is less than 70 W and the TWT is conduction cooled through mounting plate and air fan, which makes the device capable of operating in continuous wave mode. A Pierce’s electron gun and periodic permanent magnets are employed. Chemical vapor deposition diamond disc is used in the input and output radio frequency (RF) windows to minimize the loss and voltage standing wave ratios of the traveling wave tube. Double stages deeply depressed collector is used for improving the total efficiency of the device, which can be over 5.5% in band. The weight of the device is 2.5 kg, and the packaged size is 330 mm × 70 mm × 70 mm.

## 1. Introduction

G-band electromagnetic wave provides availability for the design of terrestrial and satellite radio communication networks according to the radio regulations of International Telecommunication Union.

The European Commission Horizon 2020 ULTRAWAVE, “Ultra-capacity wireless layer beyond 100 GHz based on millimeter wave Traveling Wave Tubes”, aims to exploit portions in the millimeter wave spectrum for creating a very high-capacity layer [1].

However, there are two problems. One problem is the atmospheric attenuation, which directly influences using these frequencies for long range communication [2]. The high atmosphere attenuation and the lack of enough transmission power limit the range to a few tens of meters, even by using high gain antennas [3].

Another problem is that there is a frequency range called “Terahertz Gap” between the highest frequency of microwave technology and the lowest frequency of photonic technology [4]. 

Vacuum electronic devices (VEDs) exhibit the advantages of high average power, high operation frequency, and high efficiency. Traveling wave tubes (TWTs), one of the most widely used VEDs, exhibit the incomparable advantage of wideband, which is much higher than that of the available solid-state devices [5]. In THz regime, TWTs are the most widely used VEDs.

In recent years, great progress has been made in the development of G-band TWT. Some TWTs operating at G-band have been demonstrated [6,7,8,9,10,11], and the performances of the TWTs are shown in Table 1.

The bandwidth of most of the TWTs have narrower bandwidth (≤10 GHz), and half of them cannot operate at continue wave (CW) mode. These performances of these devices limited the applications in wireless communications, which require wider bandwidth (3 dB bandwidth ≥10%), higher operational duty cycles (100%), higher total efficiency (≥5%), higher gain (≥30 dB), and lower gain ripple (≤10 dB in band).

In order to solve the problems of the above G-band TWT and fit the requirements of terahertz communication applications, a G-band wideband continuous wave TWT is designed by Beijing Vacuum Electronics Research Institute (BVERI) and described in this article. The device is developed according to the following design routes, which is different from normal.

(1) In order to realize wideband beam–wave interaction, phase shift beyond 540° is used as the working points, where the coupling impedance is low, but the dispersion is flat.
(2) The highest frequency (230 GHz) in band is used as the reference frequency instead of the center frequency, which is beneficial to the optimization of structure parameters and electrical parameters to reduce the gain ripple.
(3) A double stages deeply depressed collector is used for improving the total efficiency of the TWT.

By the above design routes, a G-band TWT with a continuous wave output power of 8 W and a gain of 30.5 dB with 27 GHz bandwidth is realized. The maximum output power is 16 W and the bandwidth of 10 W output power is 23 GHz. The 3 dB bandwidth is greater than 12.3% of f_c_ (center frequency). The gain ripple is less than 10 dB in band.

## 2. TWT Design and Simulation

**Figure 1:**
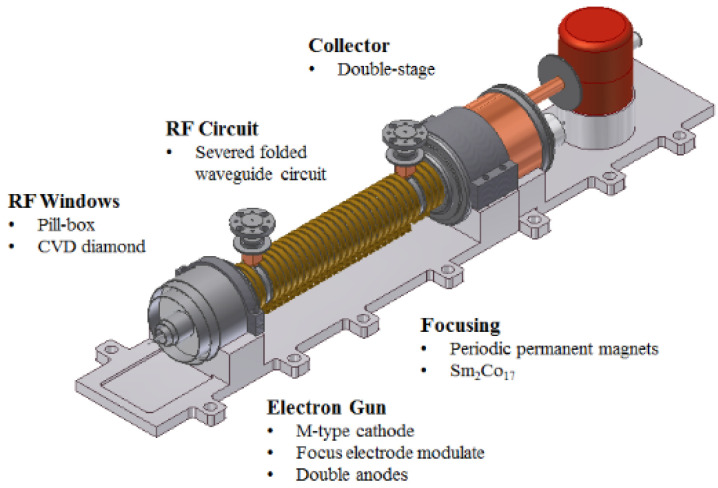
Building blocks of the G-band TWT.

The TWT primarily contains five parts: electron gun, focusing system, radio frequency (RF) circuit, RF windows, and collector. The building blocks of the TWT are shown in Figure 1.

**Figure 2:**
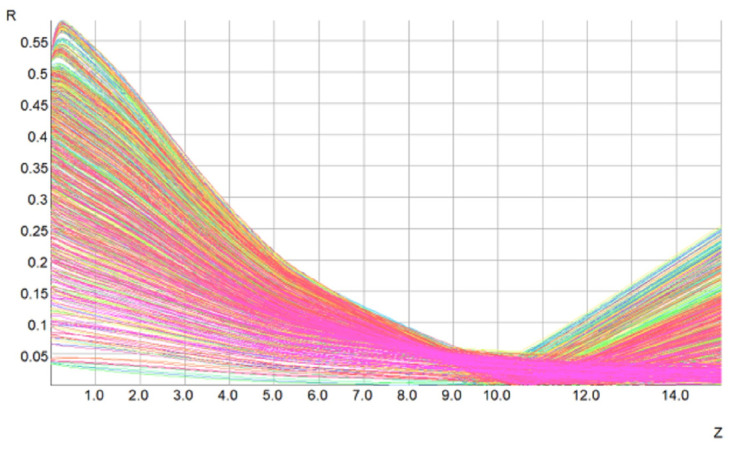
Simulation result of the electron gun.

A Pierce’s electron gun is used to produce an electron beam with a current of 20 kV and 50 mA. The type of the cathode is M-type and the cathode loading is 5 A/cm^2^. A focus electrode modulates the beam providing a duty cycle from 0.1% to continuous wave. The double anodes adjust the beam current and transmission. Opera 3D is used to design the electron gun, and the simulation result is shown in Figure 2. The designed beam voltage and current of the TWT are 20 kV and 50 mA with a beam radius of 0.06 mm and a beam-shot of 10 mm, respectively.

**Figure 3:**
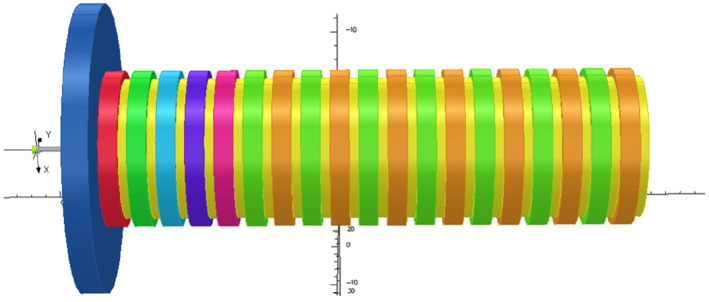
Model of Sm2Co17 periodic permanent magnet focusing system.

Sm_2_Co_17_ periodic permanent magnet (PPM) is used as the focusing system. The samarium cobalt magnets produce an on-axis *B*_z_ whose peak value is 0.5 T. The system is simulated by opera 3D. Figure 3 shows the simulation model. According to the simulation results, a high beam transmission ratio of 99% through the small diameter beam tunnel in the slow wave circuit is essential.

**Figure 4:**
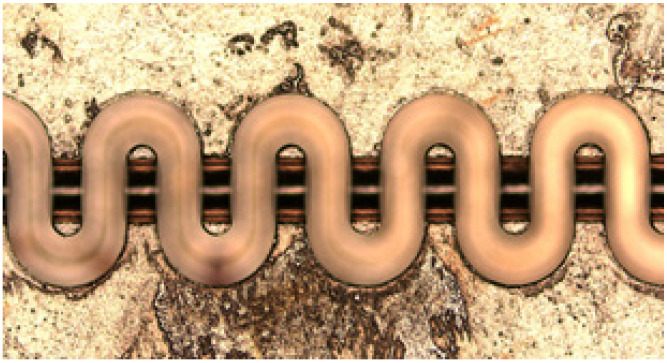
The fabricated circular of the folded waveguide slow-wave structure.

A folded waveguide is employed as the slow-wave structure in the TWT. It is fabricated with CNC-machining. The material chosen was oxygen free copper. The fabricated circular is shown in Figure 4.

**Figure 5:**
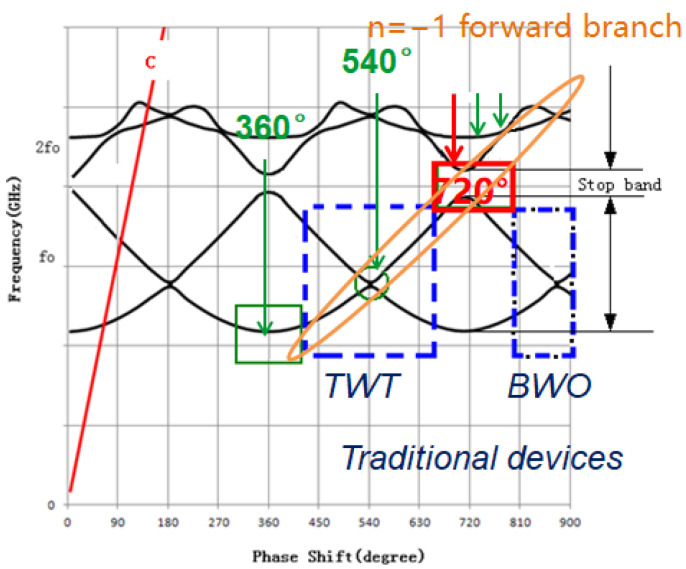
Dispersion curves of a traditional folded waveguide circuit.

Figure 5 shows the dispersion curves of a traditional folded waveguide circuit. For traditional fold waveguide slow-wave structure, *n* = −1 forward branch of the dispersion curve was used for the circuit, which operated as usual.

For the fully symmetric folded waveguide slow-wave structure, there is no stop band at phase shift of 540° in theory. However, due to the machining, the actual slow wave structure will have a certain asymmetry, which may result in a stopband at the phase shift of 540° [12]. This stopband may affect the matching characteristics at the frequency, which may depress the output power at this frequency. In addition, it may result in the undesired 3π oscillations. Therefore, phase shift of 540° is usually avoided to fall into the operating frequency band when designing the folded waveguide slow-wave structure.

According to the calculation formula of coupling impedance

$$\;K_{c}=\frac{{\left|E_{z}\right|}^{2}}{2\beta P}$$

for folded waveguide slow-wave structures, the closer to the cutoff frequency, the stronger coupling impedance is. Here, *E_z_* is the axial component of the electric field, *β* is the phase constant of the electromagnetic wave, and *P* is the power flowing. Therefore, in order to obtain greater power and higher interaction efficiency, the operating point is usually selected within 540°.

However, as shown in Figure 5, the phase shift within 540° means that the dispersion is stronger, which directly affects the operating bandwidth of the TWT. As the dispersion intensity of the folded waveguide slow-wave structure is positively correlated with the proportion of the beam injection channel size to the waveguide, due to the limitations of the performance of the current focusing system, it is difficult to further reduce the size of the beam injection channel in the THz band.

One way to achieve broadband performance is selecting the operating point above 540°, which is not usually used for its lower coupling impedance. The dispersion is more flat, which is more conducive to the synchronization and interaction between beam and wave. In addition, as the operating point moves away from the cutoff frequency, high frequency loss can be reduced. The saved power from high frequency loss can be stored in the beam and recovered by the deeply depressed collector with an efficiency of more than 90%.

At the same time, the decrease of electronic efficiency can inhibit the dynamic defocus of the beam, which is beneficial to improve the dynamic flow rate.

**Figure 6:**
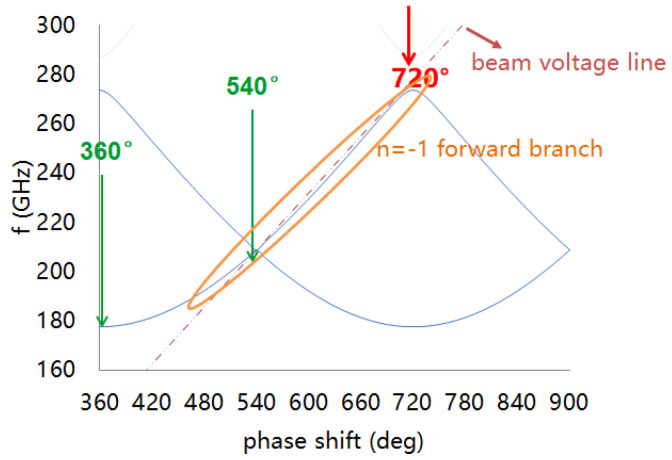
Dispersion curves of the folded waveguide circuit.

**Figure 7:**
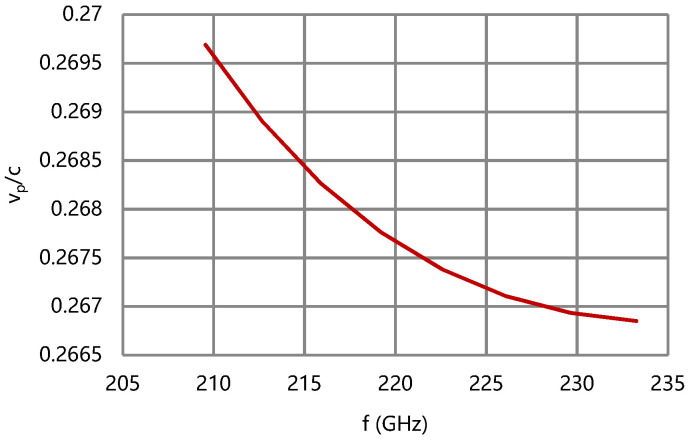
Normalized phase velocity of the folded waveguide circuit.

**Figure 8:**
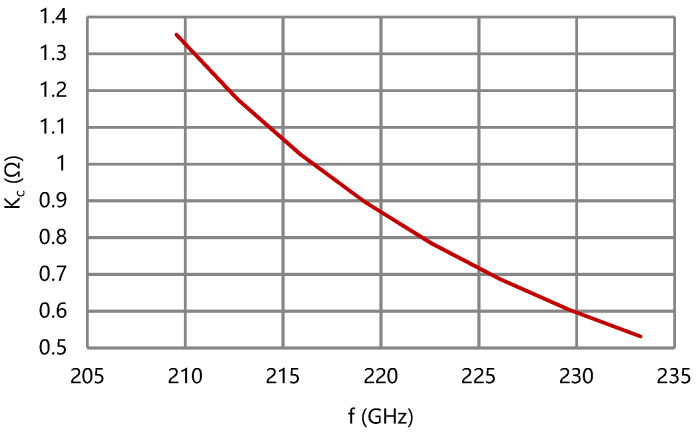
Coupling impedance of the folded waveguide circuit.

**Figure 9:**
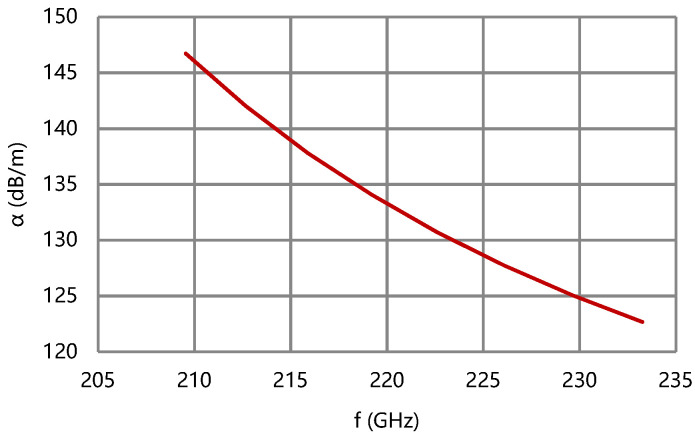
Attenuation coefficient of the folded waveguide circuit.

By the above design routes, the G-band broadband folded waveguide slow-wave structure is designed, and its cold characteristics are calculated by CST Microwave Studio. The simulation results are shown in Figure 6, Figure 7, Figure 8 and Figure 9.

Considering the effects of dispersion, coupling impedance, and attenuation, the working point is selected between 540° to 630°. The beam line almost coincides with all frequency points from 208 GHz to 233 GHz, which ensures excellent beam–wave synchronization in the band and allows a wideband beam–wave interaction.

According to the results of Figure 7 and Figure 8, the difference of phase velocity is less than 1% of v_pc_ (center) and the coupling impedance of the folded waveguide circuit is over 0.5 Ω in band. The effective conductivity of the circuit is empirically set as 2.6 × 10^7^ S/m considering the surface roughness. The attenuation coefficient of the folded waveguide circuit is less than 150 dB/m, as shown in Figure 9.

The severed folded waveguide circuit consists of an input section and an output section, which ensures the stable operation while providing a high gain over 30 dB with low ripples.

The center frequency is usually selected as the reference frequency to find the operating voltage when designing TWTs. The method is applicable when designing low frequency TWTs or THz narrow band TWTs, as their variation of in-band coupling impedance is small. However, for THz wideband TWT, the in-band coupling impedance varies strongly, and the coupling impedance of which at the low frequency may be more than three times that at the high frequency end, resulting in large gain ripple.

The design scheme in this paper does not follow the traditional design scheme, and the highest frequency in band has been taken as the reference frequency to determine the operation voltage. By adjusting the dispersion strength of the slow-wave structure, the beam–wave interaction performance at the low-frequency can be adjusted, which can bridge the gain ripple caused by the change of coupling impedance.

**Figure 10:**
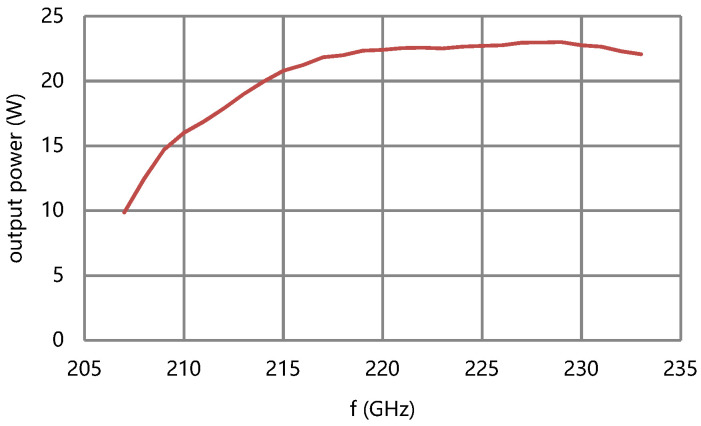
Saturation output power of the circuit.

**Figure 11:**
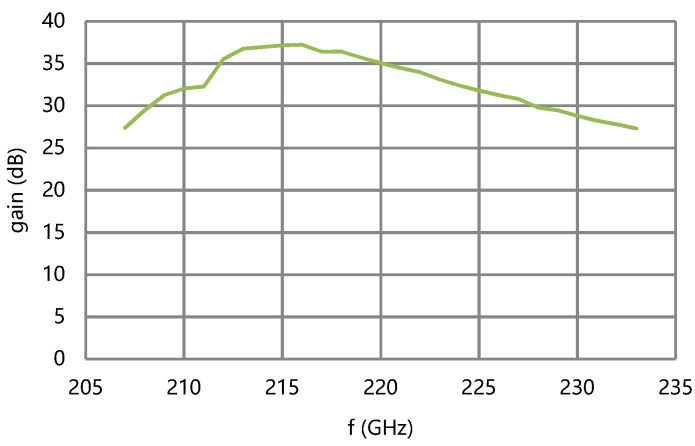
Saturation gains of the circuit.

The performance of the circuit is simulated by using a large signal beam–wave interaction software microwave tube simulator suite (MTSS). The saturation output power of the circuit is over 12 W and the saturation gain is over 27.8 dB in 208–233 GHz at the designed beam voltage and current, as shown in Figure 10 and Figure 11.

**Figure 12:**
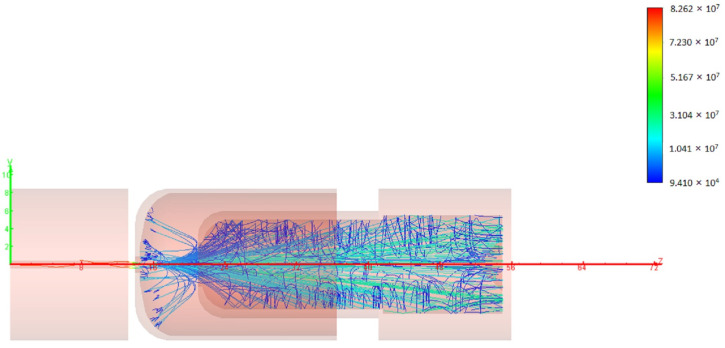
Electron distribution in the double stages depressed collector.

In order to increase the total efficiency, a double stage depressed collector with an efficiency of over 90% is used in the TWT. The design voltage of the first stage is 17.5 kV, and the voltage of the second stage is 18.5 kV. The simulation results are shown in Figure 12 and Table 2. According to the results, the total efficiency of the TWT can be over 8% in band.

**Figure 13:**
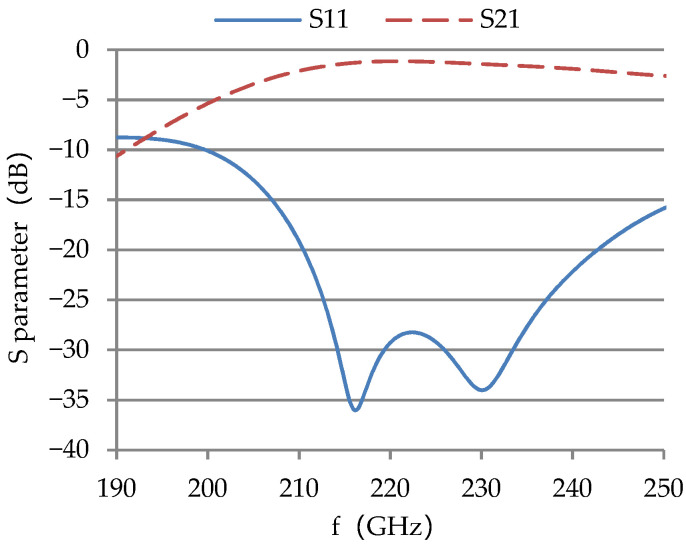
Measured S11 and S21 of a typical RF window employed in the TWT.

Diamond is used as window disk material because of its small dielectric constant, small loss tangent, high thermal conductivity, and good broadband matching. Both the input and output RF windows of the TWT are pillbox windows, and the waveguide standard WR-4 is selected according to the operating frequency. CST Microwave Studio was used to optimize the S-parameters of the window. The measured S_21_ of a typical RF window is about −1 dB and the S_11_ is lower than −10 dB in 200–240 GHz, as shown in Figure 13.

## 3. TWT Performance

**Figure 14:**
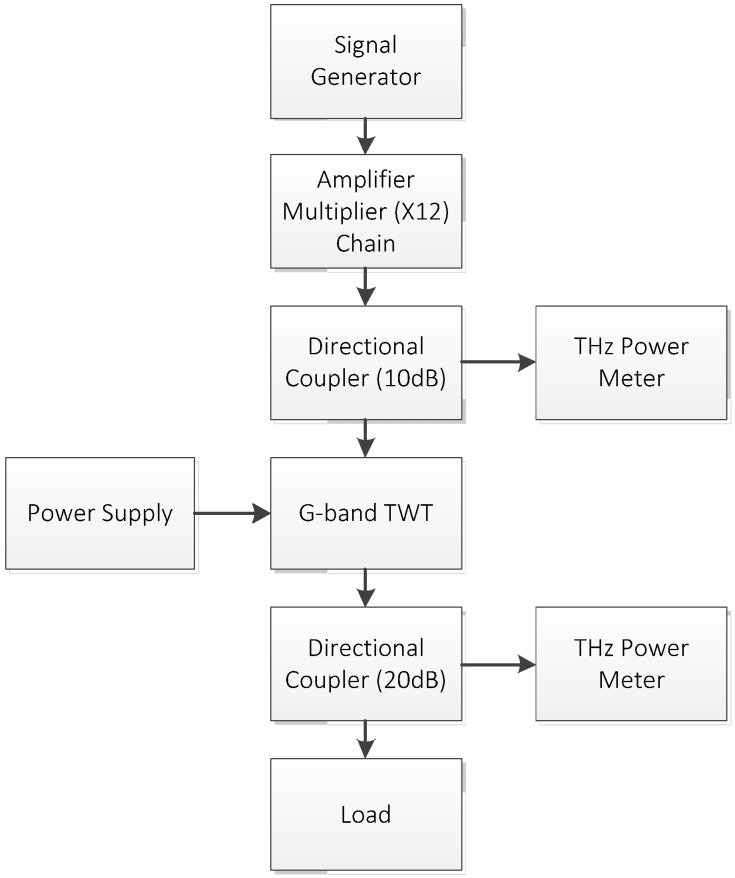
Block diagram of the experimental setup.

**Figure 15:**
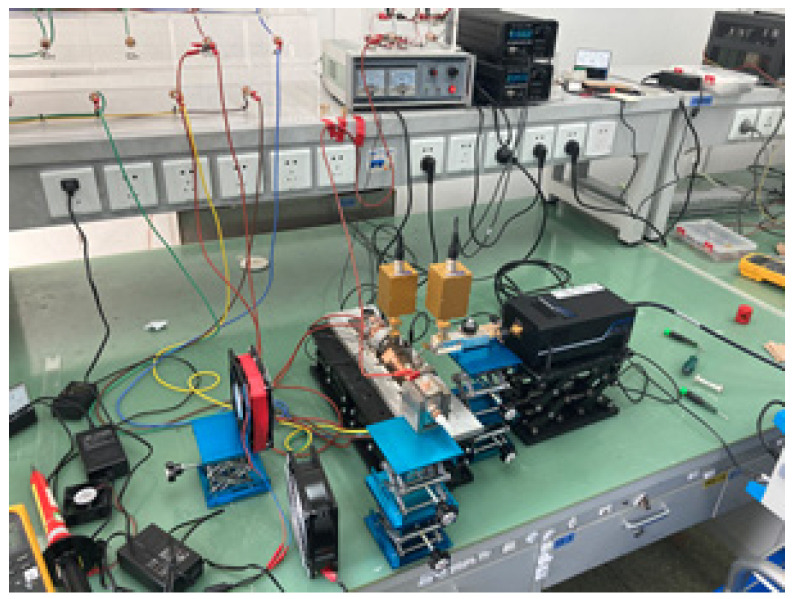
Test system.

The block diagram of the experimental setup is shown in Figure 14, and the test system is shown in Figure 15. A solid-state amplifier-multiplier chain (AMC) is used to provide the input power for the TWT. Two directional couplers are used to sample the input and output power of the TWT, respectively. The input and output power are measured with two THz power meters simultaneously.

The TWT operates in continuous wave mode at an optimal voltage of 20 kV and a beam current of 50 mA. The body current is 3 mA without RF and the worst body current with RF is 3.5 mA. The corresponding electron transmission ratio is over 93%. The TWT is conduction cooled through the mounting plate.

**Figure 16:**
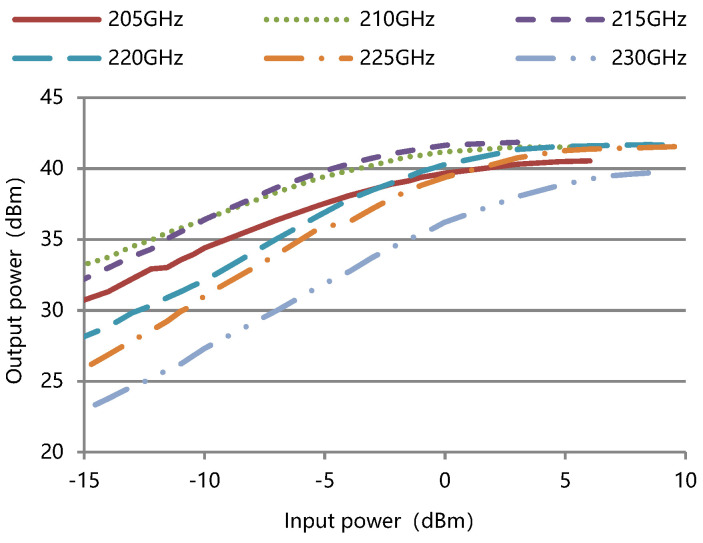
Measured output power against input power for the TWT at different frequencies.

**Figure 17:**
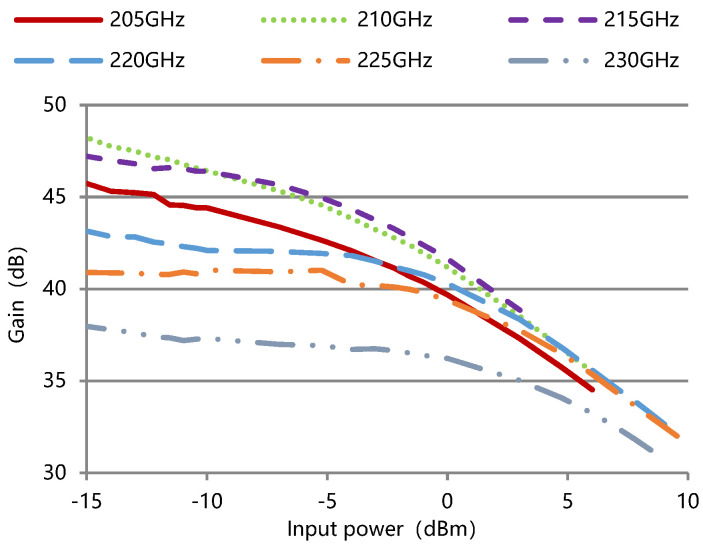
Measured gain against input power for the TWT at different frequencies.

The measured output power and gain against input power for the TWT at different frequencies are shown in Figure 16 and Figure 17. The input and output segments of the slow-wave structure have the same size in the design, and the AM (amplitude modulation) /AM was not specifically considered. We plan to use anomalous dispersion to improve linearity in the future.

**Figure 18:**
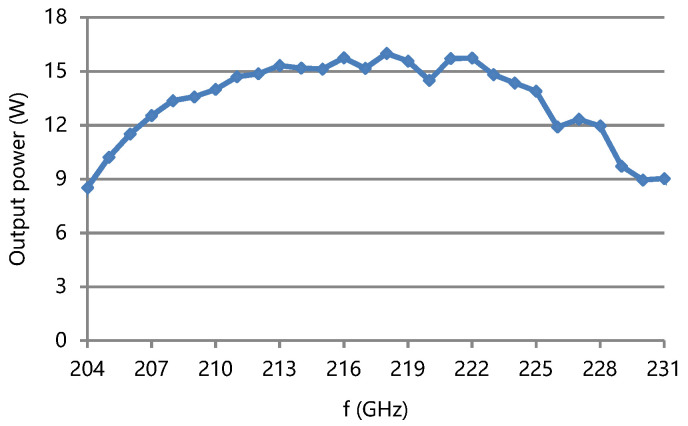
Measured saturation output power of the TWT.

**Figure 19:**
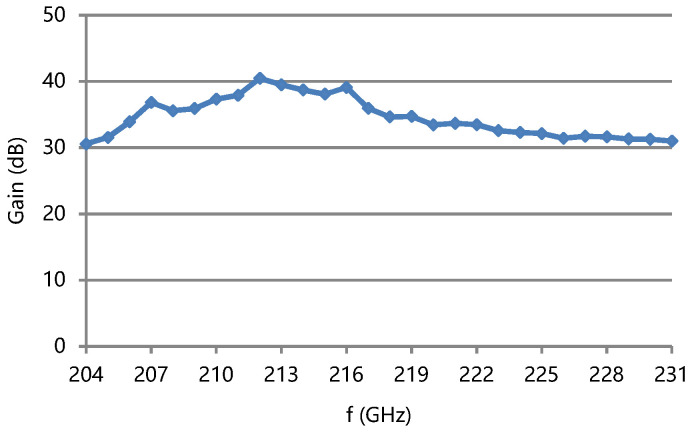
Measured gain of the TWT.

The measured saturation output power and gain of the TWT are shown in Figure 18 and Figure 19. The saturation output power is over 8 W and the saturation gain is over 30.5 dB in 204–231 GHz. The saturation output power is over 10 W in 205–228 GHz. The maximum output power is 16 W at 218 GHz. The 3 dB bandwidth is greater than 12.3% of f_c_. The gain ripple is less than 10 dB in band.

Comparing the measured saturation output power of the TWT with the simulation results, the output power is lower than simulated. One reason for this is that the beam current is set as 50 mA in the simulation, however, electrons of 3.5 mA are intercepted before they research the output port of the TWT, which can depress the beam–wave interaction efficiency. Another reason is that the insertion loose of the RF window is estimated as 1.5 dB in the simulation, which has been verified by the cold test of the RF window.

In addition, the maximum output power of the solid-stage source is less than 8 mW beyond 228 GHz. This is why the output power beyond 228 GHz reduced significantly.

Comparing the measured gain of the TWT with the simulation results, the gain is higher than simulated. A possible reason is that the input beam is thicker in the input part than design. This can let the average coupling impedance in the beam cross section be higher than the design. Stronger beam–wave interaction can occur, and the gain is higher.

**Figure 20:**
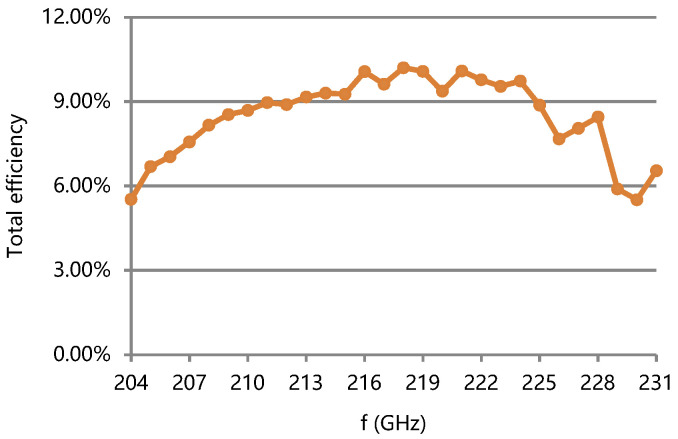
Measured total efficiency of the TWT.

The maximum efficiency of the TWT can research 10.2% and the total efficiency is over 5.5% in band, corresponding to a power dissipation of less than 160 W, as shown in Figure 20.

**Figure 21:**
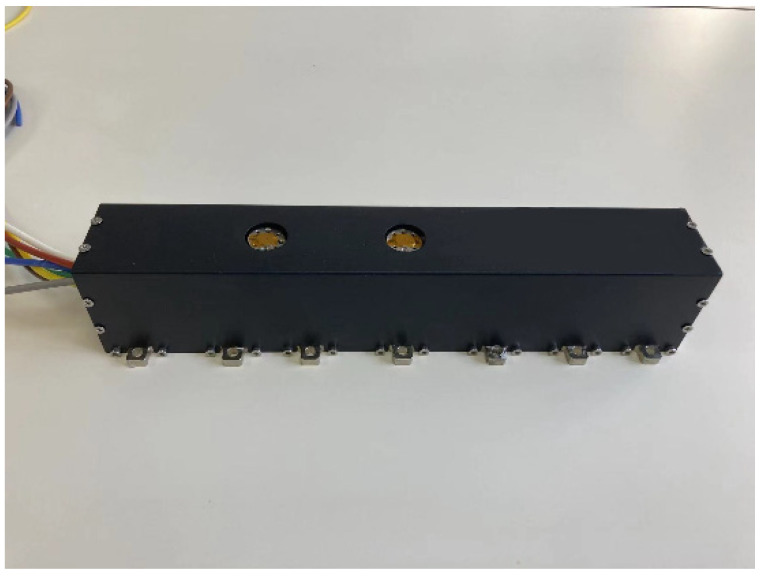
The photo of the packaged G-band TWT.

Figure 21 is the photo of the packaged G-band TWT. The weight of the packaged TWT is 2.5 kg and the size is 330 mm × 70 mm × 70 mm, respectively.

## 4. Conclusions

This article presents the development of a G-band broadband continuous wave TWT for wireless communications based on a slow-wave structure of fold waveguide. The device provides the saturation output power over 8 W and the saturation gain over 30.5 dB with a bandwidth of 27 GHz. The maximum output power is 16 W and the bandwidth of 10 W output power is 23 GHz. The 3 dB bandwidth is greater than 12.3% of f_c_. The gain ripple is less than 10 dB in band. A pencil beam of 50 mA and 20 kV is used and a transmission ratio over 93% is realized. A double stages deeply depressed collector was used for improving the total efficiency of the device, which can be over 5.5% in band. The weight of the device is 2.5 kg, and the packaged size is 330 mm × 70 mm × 70 mm.

## Figures and Tables

**Table 1:** Some G-band TWTs performances in recent years.

No.	Output Power	Bandwidth	Gain	Operational Duty Cycles	Organization
1	50 W	2.4 GHz	—	50%	Northrop Grumman
2	50 W	3.6 GHz	35 dB	5%	BVERI
3	11 W	14 GHz	27 dB	—	UC Davis
4	9 W	10 GHz	25 dB	CW	China Academy of Engineering Physics
5	14.1 W	7 GHz	30.7 dB	CW	China Academy of Engineering Physics
6	15 W	7.6 GHz	32 dB	CW	BVERI
7	8 W (3 dB)	27 GHz (12.3% f_c_)	30.5 dB	CW	This TWT
	10 W	23 GHz			

**Table 2:** The simulation results of different frequency.

f (GHz)	Collector Efficiency (%)	Total Efficiency (%)
210	91.73	8.03
220	91.38	9.5
230	91.99	8.15

## Data Availability

Not applicable.
